# Letter to the Editor response: Nygaard *et al*.

**DOI:** 10.1093/biostatistics/kxw031

**Published:** 2016-10-25

**Authors:** F. Towfic, R. Kusko, B. Zeskind

**Affiliations:** *Immuneering Corporation, One Broadway, 14th Floor, Cambridge, MA 02142, USA*bzeskind@immuneering.com

## Abstract

The article by Nygaard *and others* (2016) proposes that applying batch correction approaches to microarray data from studies with unbalanced designs may inadvertently exaggerate the differences observed. In seeking to illustrate their point, Nygaard *and others* (2016) utilized a dataset (GSE61901) from a study we published (Towfic *and others*, 2014) and showed that one analysis pipeline utilizing the traditional approach to batch correction (ComBat) yielded over 1000 differentially expressed probesets, while an alternative approach proposed by Nygaard *and others* (2016). (utilizing batch as a fixed effect and averaging technical replicates) recovered 11 differentially expressed probesets.

The article by Nygaard *and others* (2016) proposes that applying batch correction approaches to microarray data from studies with unbalanced designs may inadvertently exaggerate the differences observed. In seeking to illustrate their point, Nygaard *and others* (2016) utilized a dataset (GSE61901) from a study we published (Towfic *and others*, 2014) and showed that one analysis pipeline utilizing the traditional approach to batch correction (ComBat) yielded over 1000 differentially expressed probesets, while an alternative approach proposed by Nygaard *and others* (2016; utilizing batch as a fixed effect and averaging technical replicates).

recovered 11 differentially expressed probesets.

While we agree with Nygaard *and others* (2016) premise that the utilization of ANOVA or empirical-Bayes derived approaches to correct for batch effects may in certain cases overestimate significance, the approach they propose is surprisingly sensitive to differences in how data are preprocessed, specifically the handling of technical replicates. We therefore sought to identify alternate methods that address the points raised by Nygaard *and others* (2016). These efforts yielded three alternative approaches for analyzing microarray data from studies with similar designs to ours, each of which adjusts for technical variation while avoiding the risks that Nygaard *and others* (2016) described:

(i) Utilize the technical replicates as a random-effect variable in LIMMA.
(ii) Utilize the technical replicate as a random variable in a repeated-measures ANOVA.
(iii) Utilize a linear mixed-effects model to account for the technical replicate as a random effect while accounting for treatment as a fixed effect.

Each of the aforementioned methodologies is readily accessible through R’s base or Bioconductor packages.

Nygaard *and others* (2016) referenced a study (GSE61901) we conducted (Towfic *and others*, 2014), in which we found significant and biologically relevant differences between the gene expression profiles induced by glatiramer acetate (GA, Copaxone), and the gene expression profiles induced by a generic GA (Glatimer$^{\mathrm{\circledR }}$, Natco Pharma, Ltd., Hyderabad, India) using Illumina$^{\mathrm{\circledR }}$ Mouse-WG6 microarray chips. Each WG6 chip contains six microarrays, and each of the microarrays containing two physical strips that are to be treated as technical replicates (Shi *and others*, 2009). Because the distribution of the samples on each chip was heavily unbalanced (see supplementary Table 1 available at *Biostatistics* online), the dataset was initially analysed with ComBat including the treatments as covariates as required by the SVA package (Leek *and others*, 2012) and noted by Nygaard *and others* (2016). The dataset was further analysed using various methods including two-way ANOVA and LIMMA yielding hundreds of differentially expressed probesets between GA and generic.

**Table 1.** *Number of differentially expressed probesets after FDR $<$ 0.05 cutoff using (1) averaging technical replicates without using ComBat (2) ComBat $+$ statistical analysis (common pipeline) (3) accounting for technical replicates using mixed effects models (advanced pipeline). As can be seen from the table, the common pipeline of quantile normalization $+$ ComBat and the advanced pipeline of utilizing mixed effect models clearly detect more significant changes after FDR adjustment compared to models suffering from low power due to (1) averaging of technical arrays (2) Not utilizing a batch model that preserves power prior to analysis*

	Quantile normalization (averaging technical replicates), utilize CHIP as blocking variable	Quantile normalization (averaging technical replicates), apply ComBat including treatments as covariates	Quantile normalization, no averaging of technical replicates, utilize CHIP as blocking variable
LIMMA	11 (Nygaard *and others* (2016)	974	1474
Two-way ANOVA	1	742	—
Linear mixed effects model	—	—	1968
Repeated measures ANOVA	—	—	1749

In contrast, Nygaard *and others* (2016) performed an analysis in which all the technical replicates were averaged. After averaging all technical replicates, Nygaard *and others* (2016) utilized the chip ID as a blocking variable in the analysis model, finding 11 probesets that pass FDR cutoff of 0.05. Another approach is to utilize the duplicateCorrelation feature recommended by LIMMA’s authors for handling technical replicates from Illumina WG-6 BeadChips (Shi *and others*, 2009). While averaging technical replicates has been suggested in the literature in cases where a t-test or two-way ANOVA will be utilized for downstream analysis (Cui and Churchill, 2003), the utilization of models that can account for both technical as well as biological variation is an alternative approach to maximize power of the statistical test (Chen *and others*, 2004; Cui and Churchill, 2003; Smyth *and others*, 2005). When GSE61901 is utilized and duplicateCorrelation is applied to account for the technical replicates, we find that the approach proposed by Nygaard *and others* (2016) of blocking for batch effect in LIMMA identifies 1474 differentially expressed probesets. Thus, the results of Nygaard *and others* (2016) proposed batch correction method differ dramatically depending upon how a dataset is preprocessed.

Upon further investigation, a variety of different preprocessing and batch correction methods all yield far more differentially expressed genes than the method proposed by Nygaard *and others* (2016). Table 1 reports the results of various analyses we have conducted using a variety of techniques including LIMMA (Shi *and others*, 2009, duplicateCorrelation method: Smyth, 2013; Smyth *and others*, 2005) and mixed-effects ANOVA that are specifically designed to analyze unbalanced designs without reducing power (Bernal-Rusiel *and others*, 2013; Littell *and others*, 2002; Smyth, 2014). Code for these analyses is publicly available at https://github.com/immuneering/biostat_reply. Such methods take into account correlations between repeated measurements from the same subject/biological sample. These findings strongly suggest that the differences between GA and generic are robust to changes in batch correction and analysis methodologies, and support the key conclusions of Towfic *and others* (2014).

In summary, we present three different methods for analyzing microarray data that utilize technical replicates while correcting for batch effects. These methods yield consistent results, and therefore represent robust alternatives to traditional batch correction methods in datasets subject to the concerns articulated by Nygaard *and others* (2016).

## Supplementary Material

Supplementary Data
