# Supplementary material for: Letter to the Editor response: Nygaard et al
Source: Biostatistics. 2016 Oct 25;18(2):197–9. doi: 10.1093/biostatistics/kxw031 (PMC5379915; doi:10.1093/biostatistics/kxw031)
Supplement: Supplementary Data [file kxw031_Supp.docx]

Supplementary Table 1: Distribution of samples across chips for the original study

|  | Appotex 1 | Appotex 2 | Edan | GA C1 | GA DM | GA DP | GA FD | GA Q | GA RS | GA RS HD | Manitol | MBP | Medium | Shira | TV-5010 | TV Marker |
| --- | --- | --- | --- | --- | --- | --- | --- | --- | --- | --- | --- | --- | --- | --- | --- | --- |
| B4634633002 | 0 | 0 | 0 | 0 | 0 | 4 | 0 | 0 | 1 | 0 | 0 | 1 | 0 | 0 | 0 | 0 |
| B4634633043 | 0 | 0 | 0 | 0 | 1 | 0 | 1 | 2 | 1 | 0 | 0 | 0 | 1 | 0 | 0 | 0 |
| B4634633053 | 0 | 0 | 0 | 0 | 1 | 1 | 0 | 1 | 1 | 0 | 0 | 0 | 1 | 0 | 0 | 0 |
| B4637105025 | 0 | 0 | 0 | 0 | 0 | 5 | 0 | 0 | 0 | 0 | 1 | 0 | 0 | 0 | 0 | 0 |
| B4682416019 | 0 | 0 | 0 | 0 | 0 | 4 | 0 | 0 | 2 | 0 | 0 | 0 | 0 | 0 | 0 | 0 |
| B4682416043 | 0 | 0 | 0 | 0 | 0 | 1 | 2 | 1 | 2 | 0 | 0 | 0 | 0 | 0 | 0 | 0 |
| B4682416087 | 0 | 0 | 0 | 0 | 1 | 0 | 0 | 0 | 1 | 0 | 0 | 0 | 1 | 0 | 0 | 3 |
| B4682416090 | 0 | 0 | 0 | 0 | 0 | 4 | 0 | 1 | 0 | 0 | 0 | 0 | 1 | 0 | 0 | 0 |
| B4763128059 | 0 | 0 | 0 | 0 | 1 | 2 | 0 | 1 | 1 | 0 | 0 | 0 | 1 | 0 | 0 | 0 |
| B4763128060 | 0 | 0 | 0 | 0 | 1 | 2 | 0 | 1 | 1 | 0 | 0 | 0 | 1 | 0 | 0 | 0 |
| B4763646004 | 0 | 0 | 0 | 0 | 0 | 1 | 0 | 0 | 2 | 2 | 0 | 0 | 1 | 0 | 0 | 0 |
| B4763646012 | 0 | 0 | 0 | 1 | 0 | 1 | 0 | 2 | 1 | 0 | 0 | 0 | 1 | 0 | 0 | 0 |
| B4763646026 | 0 | 0 | 0 | 1 | 0 | 1 | 0 | 2 | 1 | 0 | 0 | 0 | 1 | 0 | 0 | 0 |
| B4763646030 | 0 | 0 | 0 | 0 | 0 | 2 | 0 | 0 | 1 | 1 | 0 | 0 | 2 | 0 | 0 | 0 |
| B5216898004 | 1 | 1 | 0 | 0 | 0 | 2 | 0 | 0 | 1 | 0 | 0 | 0 | 1 | 0 | 0 | 0 |
| B5216898008 | 0 | 0 | 0 | 0 | 0 | 2 | 0 | 0 | 1 | 0 | 0 | 0 | 1 | 1 | 1 | 0 |
| B5216898012 | 1 | 1 | 1 | 0 | 0 | 1 | 0 | 0 | 1 | 0 | 0 | 0 | 1 | 0 | 0 | 0 |
| B5216898024 | 0 | 0 | 1 | 0 | 0 | 1 | 0 | 0 | 1 | 0 | 0 | 0 | 1 | 1 | 1 | 0 |
